# Anesthesia Management of a Case of Abdominal Pregnancy Discovered Intraoperatively During an Emergency Cesarean Section: A Rare and Challenging Case

**DOI:** 10.7759/cureus.97818

**Published:** 2025-11-25

**Authors:** Rasha G Ahmad, Kundan Das, Debasmitha Chakraborty, Strahil Kotsev, Nadine B Nour

**Affiliations:** 1 Anesthesiology, Latifa Hospital, Dubai, ARE; 2 Anesthesiology and Intensive Care, Latifa Hospital, Dubai, ARE

**Keywords:** abdominal pregnancy, cesarean section, ectopic pregnancy, intensive care, intraoperative finding, obstetric anesthesia, placenta in situ

## Abstract

We present the anesthesia management of a rare case of an abdominal pregnancy discovered intraoperatively in a 30-year-old primigravida at presumed full term, who presented to the emergency department at our hospital with abdominal pain and fever. The patient presented for emergency cesarean section due to fetal distress. Spinal anesthesia was given per our hospital protocol. Intraoperative findings revealed a live abdominal pregnancy, with an intact gestational sac located on the left posterior uterine aspect, involving the entire fallopian tube, ovary, and infundibulopelvic ligament. After evaluating the surgical findings, the Obstetrician decided to leave the placenta in situ due to the extensive vascular supply. During this time, general anesthesia was given, as the patient started to bleed, and she needed to be resuscitated. The patient underwent surgical and intensive care management, including major transfusion, and was discharged in stable condition. This case highlights the diagnostic and anesthetic challenges of advanced abdominal pregnancies and the importance of multidisciplinary care.

## Introduction

A case of anesthesia management of an abdominal pregnancy diagnosed only at the time of surgery, emphasizing surgical, anesthetic, and postoperative challenges, is reported. Abdominal pregnancy is a rare form of ectopic pregnancy with high maternal and fetal morbidity and mortality [1-3]. Its incidence ranges between 1:10,000 and 1:30,000 pregnancies [4,5]. An ectopic pregnancy occurs when the fertilized egg implants outside the uterine cavity; abdominal pregnancy, its rarest form (~1% of ectopic pregnancies), involves peritoneal implantation with unique vascular challenges. Diagnosis is often missed antenatally, and management becomes particularly complex when discovered intraoperatively during the cesarean section. The first case was documented in 1708, where the diagnosis was made intraoperatively after massive bleeding [6]. The mortality rate in these cases was very high; it is 7 times higher than that of other general ectopic pregnancies and 90 times higher than that of third-trimester delivery [5].

## Case presentation

A 30-year-old, unbooked primigravida, with no history of prior surgery or medical illness, presented to the emergency department at our hospital with abdominal pain and fever at a presumed full-term pregnancy (~9 months of gestation). She was hemodynamically stable, and her vital signs at admission were BP: 137/82, pulse: 94 b/min, respiration: 19 b/min, temperature: 37.4 °C (99.3 °F), and oxygen saturation (SpO2): 100%. She was febrile at the time of admission, and her hemoglobin was 8.3 g/dL. Due to signs of fetal distress, with fetal heart rate >160 b/min, she was shifted to the OT for an emergency cesarean section. The patient was NPO for six hours, and spinal anesthesia was administered as per the anesthesia protocol for such cases.

Intraoperative findings

**Figure 1:**
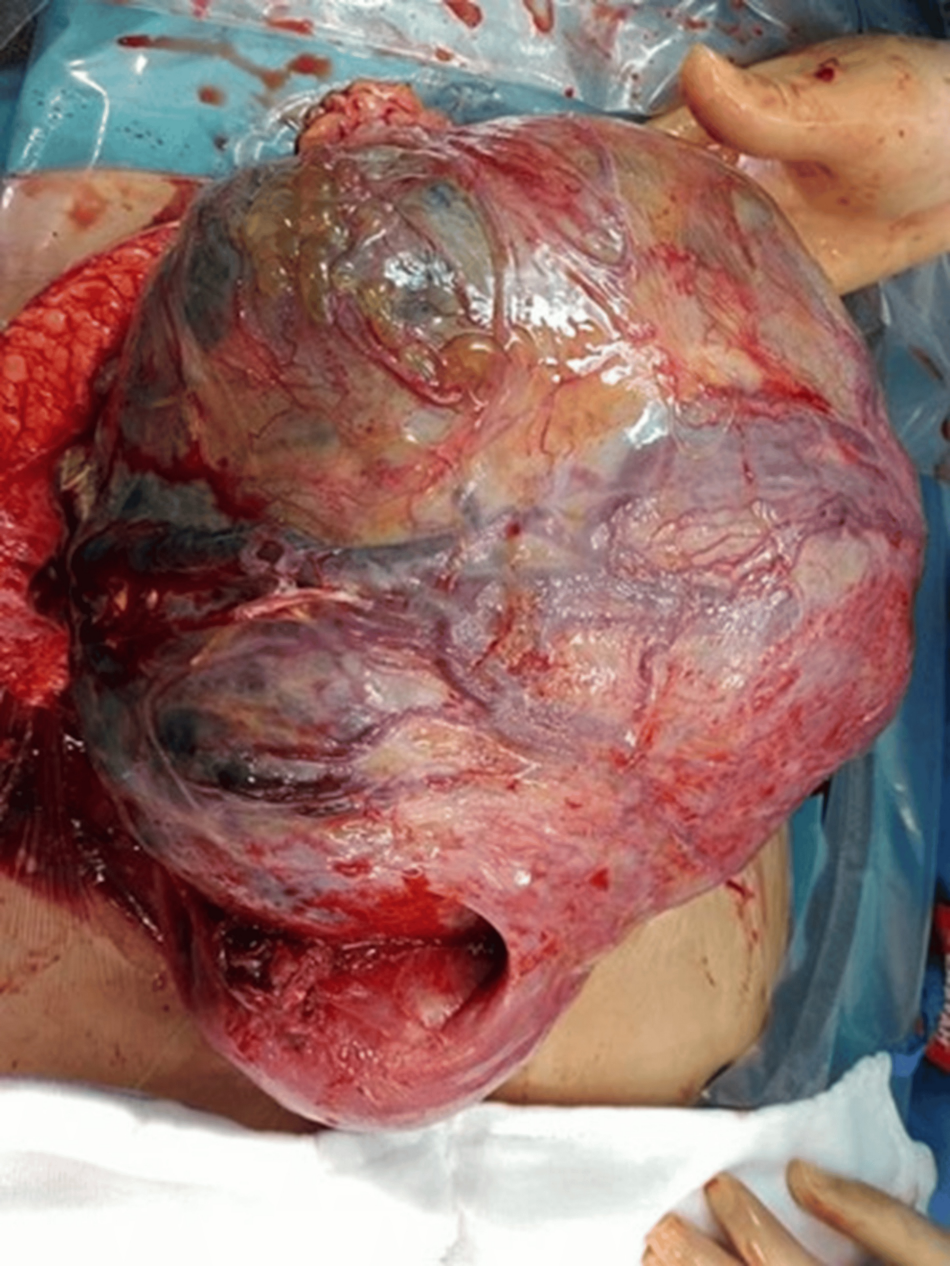
Intraoperative image showing the intact gestational sac with a richly vascularized placenta

**Figure 2:**
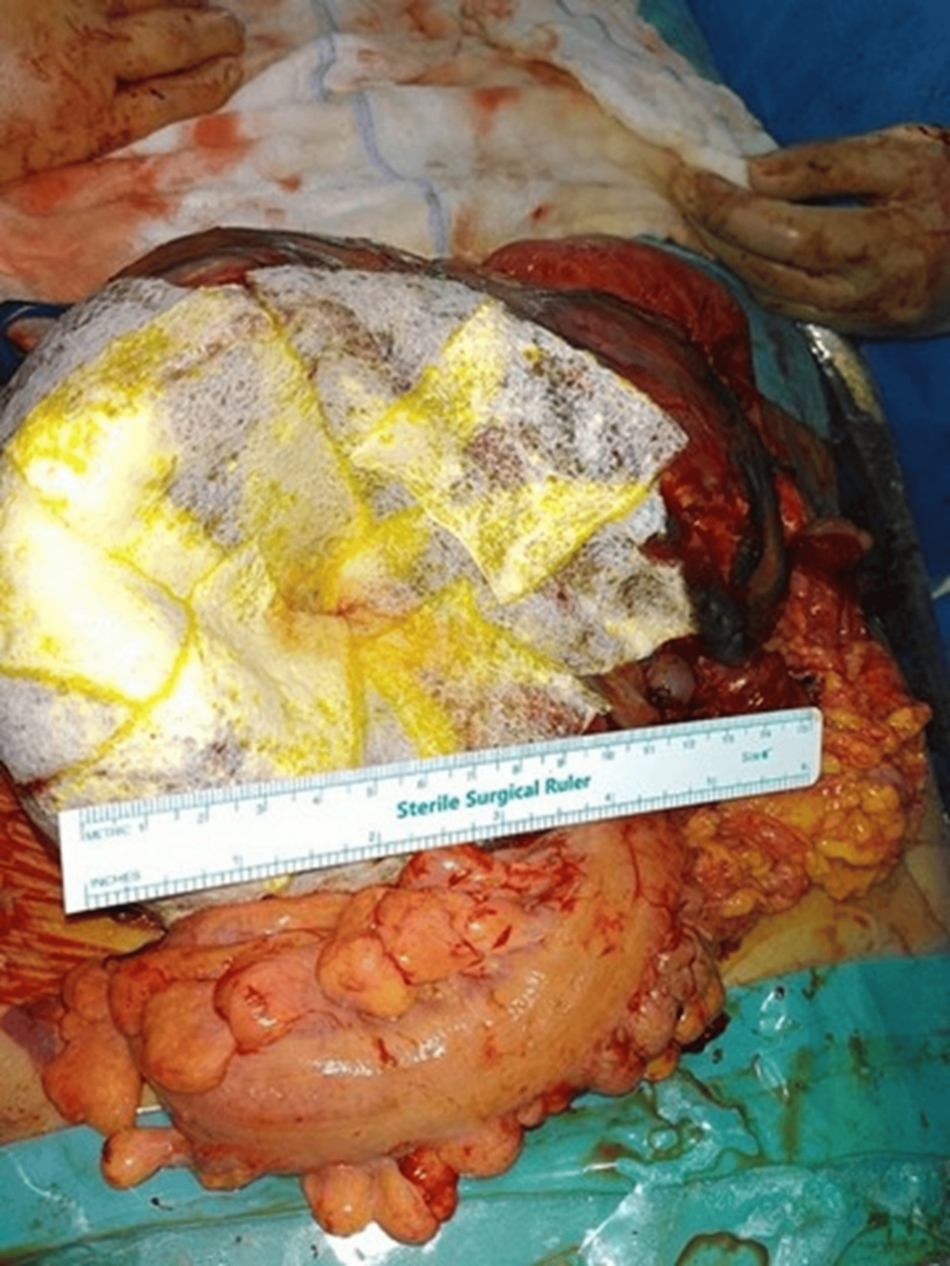
Placenta with gauze coverage to prevent bleeding and a sterile ruler for scale

After the surgery started and an incision was made, a normal gravid uterus was not identified. Instead, a large vascular abdominal mass was found occupying the pelvis and lower abdomen. The uterus was small and non-gravid. Further exploration revealed a live extrauterine fetus in an intact gestational sac attached to the left posterior aspect of the uterus, involving the ovary, tube, and infundibulopelvic ligament. A live baby girl weighing 2.5 kg with Apgar scores of 2 and 8 at 1 and 5 minutes, respectively, with no malformation, was delivered by breech extraction. The placenta was noted to be extensively vascularized and adherent to the posterior abdominal wall, bowel loops, and surrounding pelvic structures (Figures 1, 2). Due to the risk of catastrophic bleeding, a multidisciplinary decision was made to leave the placenta in situ.

Anesthetic and postoperative management

Due to the unexpected intraoperative findings and anticipated blood loss with underlying anemia, anesthesia was converted from spinal to general (spinal local anesthetic dose: bupivacaine 0.5% 11 mg + morphine preservative-free 0.1 mg + fentanyl 0.01 mg; general anesthesia induction medication: ketamine 50 mg + propofol 20 mg + suxamethonium 100 mg). Central venous and arterial lines were inserted for monitoring and resuscitation. The estimated blood loss was 1500 ml. Intraoperatively, the patient received 7 units of packed red blood cells (PRBCs) (1 uncross-matched and 6 cross-matched), 4 units of fresh frozen plasma (FFP), and 10 units of cryoprecipitate (Table 1).

**Table 1:** FBC before and after intraoperative blood transfusion FBC: full blood count; (H): high; (L): low; MCV: mean corpuscular volume; MCH: mean corpuscular hemoglobin; MCHC: mean corpuscular hemoglobin concentration; RDW: red cell distribution width; MPV: mean platelet volume

Component	Reference Range and Units	On Admission	After Transfusion
WBC Count	3.6–11.0 ×10³/µL	14.9 (H)	14.2 (H)
RBC Count	3.80–4.80 ×10⁶/µL	3.84	4.25
Hemoglobin, Blood	12.0–15.0 g/dL	8.3 (L)	11.0 (L)
Hematocrit	36.0–46.0 %	26.7 (L)	33.3 (L)
MCV	77.0–95.0 fL	69.5 (L)	78.4
MCH	27.0–32.0 pg	21.6 (L)	25.8 (L)
MCHC	31.5–34.5 g/dL	31.0 (L)	32.9
RDW	11.5–14.0 %	17.2 (H)	17.0 (H)
Platelets Count	150–410 ×10³/µL	479 (H)	202
MPV	7.4–10.4 fL	7.8	7.8
Neutrophil Absolute	2.00–7.00 ×10³/µL	11.60 (H)	12.70 (H)
Lymphocytes Absolute	1.00–3.00 ×10³/µL	2.5	0.80 (L)
Monocytes Absolute	0.20–1.00 ×10³/µL	0.7	0.7
Eosinophils Absolute	0.00–0.50 ×10³/µL	0	0
Basophils Absolute	0.00–0.10 ×10³/µL	0.1	0
Neutrophil %	—	77.8	89.7
Lymphocyte %	—	16.8	5.4
Monocyte %	—	5	4.9
Eosinophil %	—	0	0
Basophil %	—	0.4	0
Differential Type	—	AUTO DIFF	AUTO DIFF
Nucleated RBCs	0 per 100 WBC	0	0

Postoperatively, the patient was admitted to the ICU and remained intubated for 24 hours on synchronized intermittent mandatory ventilation pressure control (SIMV-PC) mode. Following major blood loss and replacement with multiple blood products, it was better to keep the patient intubated, as the risk of metabolic derangements, such as acidosis, hypocalcemia, and hypothermia, transfusion-related lung injury, and residual anesthetic effects was significant.

**Figure 3:**
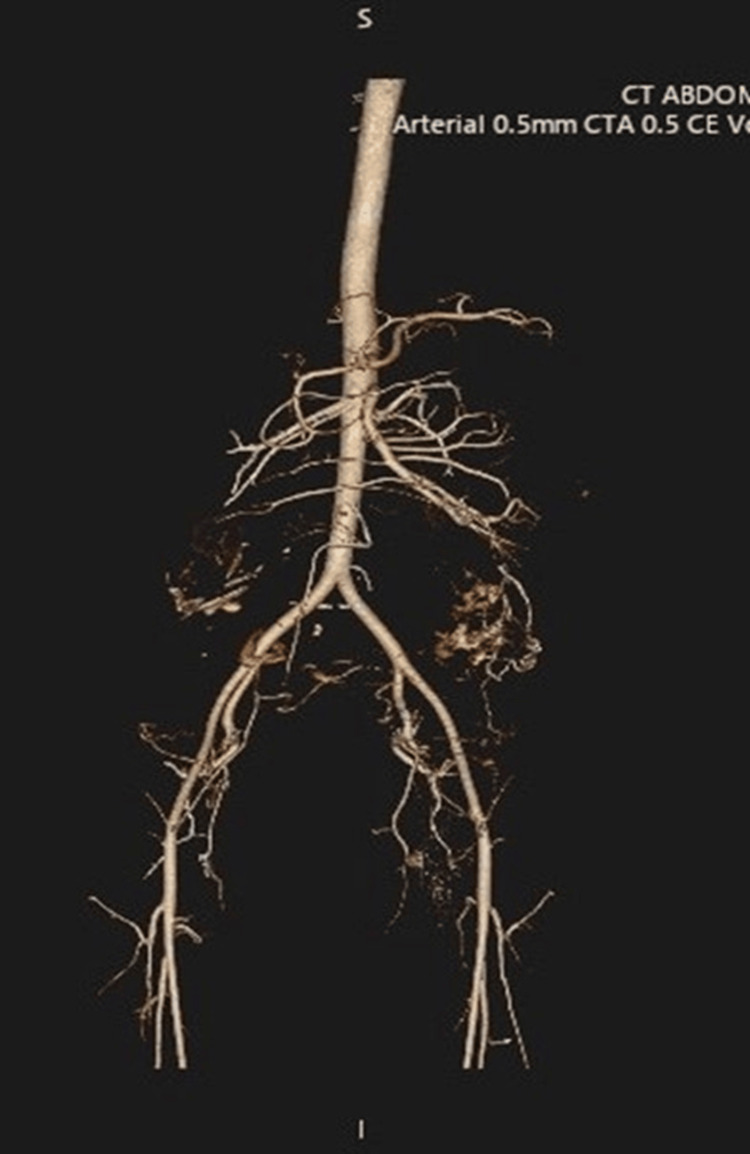
CT angiogram postoperatively shows major pelvic and abdominal vasculature feeding the placental bed

Elective postoperative ventilation allowed optimization of oxygenation, correction of coagulation and metabolic abnormalities, and close hemodynamic monitoring until the patient's condition stabilized and safe extubation criteria were met. The obstetric team was concerned about rebleeding or the need for re-exploration, so keeping the patient intubated ensured rapid access to anesthesia if urgent reoperation was needed. She received 2 additional units of PRBCs in the ICU because her Hb dropped to 9 g/dL on the second day, and she was tachycardic, on broad-spectrum antibiotics, and under close monitoring for potential bleeding and sepsis. Serial imaging with ultrasound and CT angiogram showed regression of the placental mass (Figure 3). Serial beta-hCG and infection markers were monitored until normalization.

Outcome and follow-up

The patient was gradually weaned off ventilator support and was extubated on the second postoperative day. She was transferred from the ICU to the ward after six days and continued to recover well. The total transfusion included 9 units of PRBCs, 4 units of FFP, and 10 units of cryoprecipitate. Antibiotics included IV triple therapy followed by Tazocin (piperacillin-tazobactam) and later transitioned to oral Augmentin (amoxicillin-potassium clavulanate) and metronidazole. She was discharged home in a stable condition with instructions for follow-up in her home country.

## Discussion

Abdominal pregnancy is a rare and potentially fatal condition [4-6]. It is an ectopic pregnancy where the embryo implants and grows in the peritoneal cavity, outside the uterine cavity, fallopian tubes, or ovaries. It accounts for approximately 1% of all ectopic pregnancies and is associated with high maternal and fatal morbidity and mortality due to the challenges in diagnosis and the risk of catastrophic hemorrhage, particularly when the placenta is attached to vital organs or major vessels [4,5]. It is classified into two types: 1) Primary, where the pregnancy is implanted directly into the abdominal cavity; in these cases, the Fallopian tubes and ovaries are intact. 2) Secondary accounts for most cases, where it occurs after an extrauterine tubal pregnancy that ruptures and reimplants in the abdominal cavity; in these cases, the tubes and ovaries are damaged [4,5]. Advanced cases reaching term are exceptional and pose diagnostic and management dilemmas [1-3,6-14]. The failure to identify the condition antenatally is often due to the non-specific presentation and limitations of standard obstetric imaging. Intraoperative surprise and the presence of a viable fetus challenge surgical planning and anesthetic support. The decision to leave the placenta in situ remains controversial but is often lifesaving, particularly when vascular involvement is extensive, as in this case.

In our case, the diagnosis was made intraoperatively during an emergency caesarean section. Preoperative clinical signs were nonspecific, including abdominal pain, fever, and anemia, with no prior antenatal imaging confirming an ectopic location. Despite antenatal care, abdominal pregnancies may be misdiagnosed, especially in low-resource settings or when ultrasound findings are inconclusive. This emphasizes the need for high clinical suspicion when uterine size is inconsistent with gestational age or when an empty uterus is discovered intraoperatively. The decision to leave the placenta in situ was critical in this case. Attempting to remove a placenta implanted on bowel, omentum, or pelvic vasculature can result in massive hemorrhage and high maternal mortality. In our patient, the placenta was occupying the posterior pelvic wall, covering bowel loops, sigmoid colon, and major vascular structures, which made surgical removal unfeasible. The conservative approach, supported by postoperative CT angiography and serial β-hCG monitoring, allowed safe involution of the placental tissue over time. This approach is supported in literature as a life-saving measure in similar cases [1-3]. Baffoe et al. described a term abdominal pregnancy with a healthy newborn and placenta attached to the pelvic structures, managed conservatively with favorable maternal outcomes [1]. Indrayanti et al. reported a case of abdominal pregnancy in which the placenta was left in situ due to extensive vascular involvement, also resulting in successful maternal recovery [2]. Additionally, Kim et al. described the successful anesthetic management of an advanced abdominal pregnancy, highlighting the importance of invasive line insertion and preparedness for massive transfusion [3]. These cases reinforce that in advanced abdominal pregnancies, placenta management is often the most decisive factor in maternal survival and illustrate the essential role of multidisciplinary and anesthetic planning in improving maternal outcomes.

From an anesthetic perspective, early recognition of potential bleeding risk and readiness to escalate care is vital. In this case, conversion from spinal to general anesthesia, central venous and arterial line placement, and massive transfusion support were essential components of intraoperative management. The intensive care management also included mechanical ventilation, infection control with broad-spectrum antibiotics, and serial imaging to monitor placental regression.

This case emphasizes the importance of multidisciplinary coordination among obstetrics, anesthesia, surgery, radiology, and intensive care in managing abdominal pregnancy. It also contributes to the growing body of evidence that supports conservative placental management and highlights the critical anesthetic role in maternal stabilization and perioperative decision-making. From the anesthesia perspective, it is a critical and challenging case.

## Conclusions

This case highlights the importance of maintaining a high index of suspicion for abdominal pregnancy in cases with atypical intraoperative findings, especially in the absence of a gravid uterus. Prompt intraoperative recognition, multidisciplinary collaboration, and careful anesthetic and postoperative management were essential for the favorable outcome in this rare and potentially fatal condition. Leaving the placenta in situ, although controversial, was lifesaving in this case due to the extensive vascular involvement.
